# Van Zandt County Medical Association

**Published:** 1887-04

**Authors:** 


VAN ZANDT COUNTY MEDICAL SOCIETY.—At the last meeting of this active organization Dr. W. B. Watkins of Wills Point was elected President and Dr. W. G. Johnson of Canton, Secretary.

